# From sensitization to adoption? A qualitative study of the implementation of a digitally supported intervention for clinical decision making in polypharmacy

**DOI:** 10.1186/s13012-020-01043-6

**Published:** 2020-09-21

**Authors:** Sara Söling, Juliane Köberlein-Neu, Beate Sigrid Müller, Truc Sophia Dinh, Christiane Muth, Holger Pfaff, Ute Karbach, AdAM Study Group, Petra Kellermann-Mühlhoff, Lara Düvel, Till Beckmann, Reinhard Hammerschmidt, Julia Jachmich, Eva Leicher, Benjamin Brandt, Johanna Richard, Frank Meyer, Mathias Flume, Thomas Müller, Ferdinand M. Gerlach, Ana Isabel Gonzalez-Gonzalez, Kiran Chapidi, Robin Brünn, Peter Ihle, Ingo Meyer, Nina Timmesfeld, Hans J. Trampisch, Renate Klaaßen-Mielke, Jale Basten, Wolfgang Greiner, Bastian Suhrmann, Alexandra Piotrowski, Karolina Beifuß, Sarah Meyer, Daniel Grandt, Simone Grandt

**Affiliations:** 1 Institute for Medical Sociology, Health Services Research and Rehabilitation Science, Department of Health Services Research, University of Cologne, Cologne, Germany; 2 Center for Health Economics and Health Services Research, Schumpeter School of Business and Economics, University of Wuppertal, Wuppertal, Germany; 3 Institute of General Practice, Goethe University, Frankfurt, Germany; 4 Department Sociology in Rehabilitation, Faculty of Rehabilitation Sciences, Technical University Dortmund, Dortmund, Germany

**Keywords:** Clinical computerized decision support systems, Polypharmacy, Digitalization, Evidence-based medicine, Implementation, Qualitative study

## Abstract

**Objective:**

Formative evaluation of the implementation process for a digitally supported intervention in polypharmacy in Germany. Qualitative research was conducted within a cluster randomized controlled trial (C-RCT). It focused on understanding how the intervention influences behavior-related outcomes in the prescription and medication review process.

**Methods/setting:**

Twenty-seven general practitioners (GPs) were included in the study in the two groups of the C-RCT, the intervention, and the wait list control group. Behavior-related outcomes were investigated using three-step data analysis (content analytic approach, documentary method, and design of a model of implementation pathways).

**Results:**

Content analysis showed that physicians were more intensely aware of polypharmacy-related risks, described positive learning effects of the digital technology on their prescribing behavior, and perceived a change in communication with patients and pharmacists. Conversely, they felt uncertain about their own responsibility when prescribing. Three main dimensions were discovered which influenced adoption behavior: (1) the physicians’ interpretation of the relevance of pharmaceutical knowledge provided by the intervention in changing decision-making situations in polypharmacy; (2) their medical code of ethics for clinical decision making in the context of progressing digitalization; and (3) their concepts of evidence-based medicine on the basis of professional experiences with polypharmacy in primary care settings. In our sample, both simple and complex pathways from sensitization to adoption were observed. The resulting model on adoption behavior includes a paradigmatic description of different pathways and a visualization of different observed levels and applied methodological approaches. We assumed that the GP habitus can weaken or strengthen interventional effects towards intervention uptake. This formative evaluation strategy is beneficial for the identification of behavior-related implementation barriers and facilitators.

**Conclusion:**

Our analyses of the adoption behavior of a digitally supported intervention in polypharmacy revealed both simple and complex pathways from awareness to adoption, which may impact the implementation of the intervention and therefore, its effectiveness. Future consideration of adoption behavior in the planning and evaluation of digitally supported interventions may enhance uptake and support the interpretation of effects.

**Trial registration:**

NCT03430336, 12 February 2018.

Contributions to the literature

- Digitally supported interventions have not yet been widely evaluated, and it is necessary to demonstrate effectiveness. However, great challenges are associated with obtaining insights into the complexity of adoption behavior, and little research is available in this area.
- This qualitative research synthesis study aims to methodologically and theoretically ground the Medical Research Council’s framework for the evaluation of complex interventions for obtaining an in-depth understanding of adoption behavior.
- Our analyses have shown that changes in clinical decision making about polypharmacy may occur if physicians have positive interaction experiences with the intervention, as they perceive an increased risk-awareness and willingness to base clinical decision making on scientific evidence. In comparison, physicians without digital support use habitual strategies in their daily practice to compensate for uncertainties.

## Background

A worldwide need exists for optimized and technically supported polypharmacy management in primary health care; such management systems should be based on profound evidence and prevent patient harm [1]. Even though polypharmacy is a controversial term in medical articles, it is typically associated with the use of five or more drugs and defined as a multifactorial problem of older and multimorbid patients [2]. Furthermore, it is associated with excessive and unindicated drug consumption that leads to high-risk prescription scenarios for polypharmacy patients [3]. To increase patient safety and decrease the number of potentially inappropriately prescribed medications or adverse drug events, new interventions such as technology-based management solutions have been developed, implemented, and reviewed [4, 5]. It has been demonstrated that interventions with clinical decision support systems that provide patient-specific alerts have a positive effect on prescription quality and can reduce medication errors in polypharmacy [6, 7]. In addition, there is evidence that decision aids shown on screen instead of paper-based information, as well as information provided automatically, lead to improved compliance with the recommended practice by physicians [8]. These technical solutions should enable general practitioners (GPs) to appropriately deal with high-risk prescription scenarios in polypharmacy, where they need to balance risks, benefits, and patient requests as well as avoid errors [9, 10]. Nevertheless, research regarding the implementation of health information technology is continuously reporting inconsistent effects concerning the effectiveness of technology-based interventions [7, 11].

In Germany, several health services policies have aimed to drive forward the digitalization of drug therapy safety systems and quality improvements, and many regulations are yet to be implemented in standard care [12]. In the context of the digital transformation of the German health care system, we aimed to understand primary care practitioners’ perceptions of a digitally supported intervention for improving medication safety for patients with polypharmacy.

Therefore, in this qualitative study conducted within the scope of the C-RCT project “Application for a Digitally Supported Pharmacotherapy Management System” (AdAM project—original German acronym for the project), processes leading towards adoption were analyzed. Since little is currently known about the processes through which technology-based interventions produce change and through which specific pathways lead to desired outcomes via the implementation process, our implementation process evaluation focused on this area [13]. Our research topic is in line with the Medical Research Council (MRC) framework for the process evaluation of complex interventions (2015) [14]. This framework provides one of the most promising research models for process evaluation. It proposes that a key function of process evaluation is to investigate specific mechanisms through which participants’ interactions with the intervention influence outcomes (mechanisms of impact). In accordance with the MRC framework, our study aimed to examine physicians’ behavioral interactions with the intervention and related behavior change processes.

Our research questions were the following: (1) how are clinical decision-making processes concerning patients with polypharmacy affected by the digital intervention, and (2) how does the habitus of primary care practitioners influence the adoption of the digital intervention?

## Methods

To theoretically substantiate our study, we needed to conceptualize behavior-related outcomes. Therefore, we used the definition of adoption as a phase when the decision to accept and undertake the change(s) is made [15]. “Adoption,” “usage behaviour,” or “uptake” of interventions are examples of many terms that have been used interchangeably in the field of implementation science [16]. Donaldson et al. showed that there are many theoretical approaches in implementation science that describe the same significant problem: the translation of evidence into practice [15].

The data used in this study stem from qualitative interviews and focus groups, which were collected alongside the C-RCT of the AdAM project. We investigated this topic using two different qualitative methodological approaches for data analysis: (1) We used a content-analytical approach to get an overview of the range of participants’ opinions in our study. (2) To get deeper insights into the dynamics of the change processes triggered by the intervention, we used the documentary method (interpretive methodological approach) since it is well suited for examining practical behavior-related actions and interactions [17].

An interpretive methodological approach aims to interpret qualitative data in the context of participants’ life and the interrelatedness of different aspects in life (for example individual, social, historical factors) [18]. A detailed and separate sequence analysis was conducted using a documentary methodological approach. Table 1 summarizes the methodological aspects of the study.

**Table 1:** Study design of formative evaluation

Qualitative approaches for data collection	Interviews, focus groups
Qualitative methodological approaches for data analyses	Content analysis, documentary method
Data synthesis	Process-oriented model of implementation pathways

Data saturation was reached in an iterative process. Therefore an adequate sample size was defined as one which allows sufficiently answering the research questions and includes a range of opinions [19]. The transcription of qualitative data was done by a qualified transcription office, following specific transcription rules [20]. A smooth verbatim transcription style was used. Colloquial expressions, incorrect expressions, and incorrect sentence structures were retained. Transcripts were analyzed in anonymized form. Data was coded by two researchers independently. MAXQDA was used to support data coding. The COREQ checklist was used as a reporting guideline (see Additional file 1) [21].

### Data analysis

During the iterative coding process in qualitative data analysis, it is important for (1) content analysis to find main categories under which descriptions and narrations can be subsumed and choose a level of abstraction for labeling categories (individual, social, and health care delivery level). We are speaking about the interventional influences on different levels, if descriptions or narrations of participants indicate that. The individual level is defined by us through codes related to cognitive or emotional experiences by the physicians. The health care delivery level relates to codes that include speeches about perceived changes in interdisciplinary or doctor-patient relationships caused by the intervention that might influence future health care delivery. The social level could be seen as linked to the social group of general practitioners and their perceptions of interventional influences that change their professional role (documentary method analysis).

The (2) documentary methodological approach is related to different theoretical approaches and associated with the fields of social phenomenology, ethnomethodology, and sociology of knowledge. That approach has provided specific theoretical assumptions about the evolution of collective orientations. These are important for understanding data analysis. According to Bohnsack, practical actions and interactions are guided by the habitus of social actors, who share common experience spaces and belong to similar milieus [17].

Habitus has also been defined as an organizing structure of attitudes and dispositions or “second nature,” as the way social actors behave, act, and think; it is attained unconsciously through socialization and is internalized by the actors. As Bourdieu states, practices evolve in social contexts. They can be seen as relatively autonomous, so social actors instantly understand one another if they share a habitus that guides their practical actions [22]. We therefore assume that primary care practitioners share professional experiences that are connected to a particular habitus and guide the way they behave and interact with the intervention in the implementation process.

#### Criteria for applying documentary method

The documentary method is a method of interpretation which is conducted by analyzing sequences of qualitative data (text) in a methodologically controlled way. Constitutive criteria for applying documentary method were complied with 1–3.

1. The selection process was passed through by screening of all interviews and focus groups. By consent, two sequences of a focus group (intervention group) were chosen on the basis of the relevance of content and specific text types included, for example narrations and descriptions [FG3, GP_AA-DD, p.5-6, p.22-24].
2. Interactive density of discussion during the sequences was high. By analyzing transcripts of focus group discussion, we can observe fundamental forms of sociality. The different forms of sociality are also analyzed using the methodological terminology of the documentary method. As a result, the analysis of interactive references to each other during the focus group discussion will be presented in the discourse organization [17].
3. The habitus reconstruction is a step in the process of data analysis with documentary method, in which it is examined how the same topic is dealt with in different ways by participants. Therefore, the different layers of knowledge are sequentially analyzed in the two steps of formulating interpretation (communicative or explicit meaning of talk) and reflective interpretation (conjunctive or implicit meaning of talk). Subsequent to the reconstruction of different layers of knowledge, the frames of orientation or habitus of actors can be described with the aim to understand what guides actors’ practical actions.

### Description of the intervention

The intervention evaluated in our study took place in general practices in the German state of North Rhine-Westphalia. It included multiple design components (a digitalized clinical decision support system for polypharmacy, patients’ medication history and diagnostics, information about other medical specialists, training on system use, management, and technical support for the GPs, recommendations for prescribing in polypharmacy). Patient consent allows the BARMER health insurance company to transfer actual medication data to the decision support system (medication history of the last 36 months). The study’s patient inclusion criteria were (1) prescription of five or more drugs continuously throughout the previous 6 months, (2) current insurance coverage by BARMER, and (3) adult without dementia. Signed up GPs were randomized into the wait list control group or intervention group. The external system provides, e.g., data about the patient’s diagnoses, treatment, and hospital stays, and includes an alert system for drug-drug, drug-disease, and drug-age interactions. After 15 months in the wait list control group, GPs switch to the intervention group and receive access to the software. GPs in the control group provide usual care. GPs are compensated for participation in the trial with €80 per year for each patient treated with the aid of the digital application. Concerning reporting standards, the TIDieR-checklist was used (see Additional file 2) [23].

### Participants

All contacted GPs were established doctors and provided primary outpatient care. GPs already included in the main trial received an invitation to participate in our qualitative study. The association of statutory health insurance physicians supported the recruitment process by providing the GPs with information about participating in the process evaluation study (via fax or flyer). To participate in the study, interested GPs in the intervention and wait list control group contacted our research department. We conducted interviews with the intervention group to enter the research field and get familiar with the so far made experiences of the physicians with the intervention. Focus groups were conducted with both groups of the RCT. We aimed to compare project-related expectations and experiences, depending on the participants’ C-RCT group. Participants were chosen from the RCT to evaluate the physician- and behavior-related barriers and facilitators of the implementation, which might influence the intended results of the RCT. The intervention was planned to be implemented in about 1080 practices. At the time of data collection for our qualitative study, 491 physicians were participating in both groups. Inclusion criteria were that the doctors had registered for the AdAM project and had given consent to participate in an interview or focus group with audio recording. From 36 physicians who gave us feedback for participation, 27 participants of both RCT groups were selected. The following dropout reasons were documented and represent the total number of dropouts (*n* = 9): GPs did not consent to audiotaping (*n* = 6), were not interested in participation (*n* = 1), opted out of the project (*n* = 1), or did not use the digital application (*n* = 1). All interviews with GPs of the intervention group were telephone interviews and conducted by the first author of this article (SS). Focus groups took place close to the medical practices of participating physicians, in buildings of associated medical institutions in Dortmund and Muenster. The first author of this article moderated them without the presence of non-participants. The researcher introduced herself before all interviews and focus groups and stated her professional and occupational background (health services researcher, qualified in public health and social sciences).

### Topic guides

Topic guides were used to structure the interviews and focus groups with GPs. They were created using iterative processes, applying a quality-assuring qualitative method and informed by the Consolidated Framework for Implementation Research (CFIR) [24, 25]. CFIR was used deductively and for matching the inductively developed topics. It includes a collection of important categories and a comprehensive typology in implementation research. In a workshop with a team of five multidisciplinary health services, researchers generated 17 questions related to five topics. Different topic guides for interviews and focus groups with intervention and control groups were developed and structured by main subjects and related questions. The topic guides differed in particular with regard to experiences or expectations concerning the intervention, depending on the participants’ C-RCT group and in relation to the narrative stimulus at the beginning of the focus group. Narrative stimulus in the intervention group invited participants to prioritize important experiences in the interaction with the intervention. In the control group, participants were invited to prioritize important expectations of upcoming changes related to the intervention. They were applied to gain a deeper understanding of the polypharmacy management-related health care processes that GPs employed in everyday practice. We considered the topic guides an essential narration stimulus for our research focus on the understanding of participants’ perspectives. The following five topics were chosen for process evaluation and qualitative data collection: (1) participants’ experiences or expectations towards the AdAM project; (2) GPs’ current stage of health care and polypharmacy management [26]; (3) GPs’ perceptions of interdisciplinary and doctor-patient cooperation in polypharmacy management; (4) GPs’ perceptions of the usability of the digitally supported intervention [27]; (5) organizational culture in the GPs’ practices [28–30].

## Results

In total, 27 GPs were included in our study, 15 of which were in the intervention group and 12 in the wait list control group. Table 2 shows participant characteristics as well as the average length (with range) of interviews and focus groups. From May through September 2018, in the first year of the implementation, participants were included in the evaluation study. Meanwhile, the overall recruitment process for the inclusion of GPs in the AdAM project was ongoing.

**Table 2:** Characteristics of participating GPs

Intervention group			Wait list control group	
	Interviews	Focus groups (*n* = 2)	Focus groups (*n* = 2)	Total
Number of participants	8	7	12	27
Female % (*n*)	25% (2)	43% (3)	67% (8)	48% (13)
Male % (*n*)	75% (6)	57% (4)	33% (4)	52% (14)
Duration in minutes (min)/hours (h) average (range)	24 min (10–47)	1.21 h (1.15–1.27)	1.16 h (1.11–1.20)	

### Results of content analysis

Content analysis revealed four general outcomes in both C-RCT groups. They applied to different stages of behavior-related outcomes on the individual level and the health-care delivery level. The behavior-related outcomes mentioned in stage 1 are sensitization to risks related to polypharmacy (a.1) and perceived changes of interdisciplinary and doctor-patient cooperation (b.1). In stage 2, the behavior-related outcomes mentioned are learning effects through using the digital tool (a.2), and overall perceived changes in doctor-patient communication are observed (b.2).

### Physicians’ views of interventional changes in stage 1

An especially prominent topic mentioned by participants was an emphasis on ideas of sensitization to risks related to polypharmacy. As they saw it, through increased transparency it would be possible to reflect on prescription practices and interdisciplinary or doctor-patient relationships in standard care (a.1).

You get a little more sensitive about the interactions, especially when it comes to specialist medication that you often don’t have on your radar. [ … ] If the patients don’t tell us that they are getting the medication, then we don’t know either. [FG4, GP_CC, p.14]

I think it’s good that polypharmacy is coming into focus. That doctors are sensitized to it, or that everyone is sensitized to it, and patients are also sensitized to it, and it is still a bit difficult to really get down get down from ten to five [drugs], I don’t always see myself in a position to do that, but I think it is important to be more involved than in the past ten years. And the goal is really, yes, maybe less is more. [FG2, GP_DD. p.24]

Participants described a vision where better conversations, grounded on an overview of patients’ medication history, would allow better care to be created. Achieving this would mean providing patients with evidence-based explanations on their medication, and for general practitioners to rethink interdisciplinary work with pharmacists, who are consistently identified as important experts (b.1).

So, they [patients] feel safer and also, I think, more confident about why they take something. Because you can explain what the tablets are really good for. [GP7, p.4]

I know it otherwise, as I said, also from the pharmacists, because I constantly or conveniently get information from them, like there is an incompatibility with azithromycin or something else. But where we have a comprehensive medication list from all kinds of doctors who have treated the patient, that has not yet existed. [FG3, GP_BB, p.8]

### Physicians’ views of interventional changes in stage 2

Participants expressed strong consensus on perceived learning effects triggered by the intervention: to use new, digitally enabled information on polypharmacy increases transferable knowledge into practice and changes dynamics in risky prescription scenarios. Especially the overall aim to facilitate better partnerships between actors in the communication processes related to polypharmacy prescriptions was mentioned (a.2).

I like to use it [digital tool] and see also a lot of sense in it, because I also learn again, refresh again, knowledge that is perhaps still present somewhere in the back of my mind, but to update this again, but I find this information very good.[ … ] It makes my work as a doctor much easier when prescribing, so I think that makes a lot of sense. [GP1, p.6]

I now find myself with my patients, well, coming to their routine visits, simply perceiving these risks more intensely and then changing it, yes, with the other patients as well, if I consider it initiated. And I found that, for example, quite good. [FG3, GP_DD, p.9]

Looking at the data together during routine visits was specifically intended to improve communication processes for individual patients. The information generated by the digital intervention was also used for initiating medication reviews with specialists (b.2).

I have patients where the medication just did not really fit and where I can exchange views with the specialists, who are also named [in the digital tool], where patients are being treated. [FG4, GP_CC, p.5]

It’s good, especially for the patients, they all saw great sense in it and found it good. So, I did that mostly in the presence of the patients, so they immediately saw what kind of information there was about interactions. [GP1, p.2]

The findings by the two RCT groups were similar concerning the awareness of high-risk prescription scenarios of patients with polypharmacy and reflections on changes of professional responsibilities when using digital support for decision-making. The findings differed with regard to expectation of interventional effects. Participants in the control group expressed stronger expectations of the intervention and its effects. An additional data file shows more quotes related to interventional changes in different stages (see Additional file 3). The results of the content analysis will be used in the following to be able to interpret the connections between individual, social, and interprofessional factors in the implementation process and to understand the context in which the habitus works and can be interpreted (documentary method analysis).

### Results of the documentary method analysis (formulating interpretation)

The presented core sequence analysis builds the reference point for comparisons between different text passages in our study. Different forms of sociality and the interactive references to each other during the focus group discussion are presented in the discourse organization (Table 3).

**Table 3:** Core sequence analysis

Major topic: habitus of primary care physicians	
Formulating interpretation	Reflecting interpretation (discourse organization)
Subtopic 1: medication review as a professional task of pharmacists	Proposition: introduction of a new frame of orientation
Subtopic 2: balancing effort and usefulness of the digital intervention	Elaboration in the mode of a description with modifying extension
Subtopic 3: amount of information provided by the digital intervention	Background construction in the mode of exemplification with argumentative insertion
Subtopic 4: deprescribing after hospital discharge	Validated elaboration of exemplification in the mode of differentiation
Subtopic 5: evidence-based clinical decision-making vs “healing art”	Opposition in the mode of argumentation
Subtopic 6: long-term medication and acute events	Differentiation in the mode of exemplification

At first sight, the formulating interpretation reveals *what* GPs are discussing. It is structured by topics. The introductory subject and proposition contains the description of medication review as a professional task of pharmacists (subtopic 1). In the course of the discussion, the following additional subtopics were identified: (2) balancing the effort and usefulness of the digital intervention; (3) amount of information provided by the digital intervention; (4) deprescribing after hospital discharge; (5) evidence-based clinical decision making vs. “healing art”; (6) long-term medication and acute events. In the next step, the way *how* GPs are discussing these topics is considered. The core sequential analysis with a documentary method approach furthermore demands analyzing the dynamics of interactions between participants during the discussion. *How* the discussion is organized is reflected (discourse organization) and the primary care habitus (re-)constructed.

### Results of the documentary method analysis (reflecting interpretation)

In the interplay of the sequence, the ambivalent attitudes of GPs towards evidence-based practices are manifested (subtopics 1–6). *How* the GPs discuss their usual deprescribing practice after hospital discharge documents implicitly a resistance towards integrating external evidence in their decision making (subtopics 4 and 5). Their practical actions are focused on reaching quick decisions on deprescribing based on their professional experience and without a need for external evidence.

In the transitional phase of the implementation of the new digital intervention, previous experiences with evidence-based guidelines are discussed. GPs perceive guidelines as contradictory and not applicable to medical practice in primary care (subtopic 5).

In this context, *how* primary care physicians can “heal” was discussed in comparison to medical specialists (e.g., surgeons), in a juxtaposition of physicians’ different voices and introduced topics. Medical specialists were described as a positive counter-horizon in comparison to primary care physicians because they routinely applied informed consent standards in therapeutic interventions. GPs discussed whether this practice should be transferred to prescribing practices in primary care settings in order to promote safer prescribing in polypharmacy and to share responsibility with the patient. The discourse organization shows reciprocal increase and promotion, with the dramaturgical climax being reached with the “medical healing” topic (subtopic 5).

From this finding, the generic principle of the primary care habitus—the way GPs cope with the integration of external evidence-based information from the intervention—can be derived. Guidelines for polypharmacy and included external evidence-based information are negatively connotated, and integration into practice generates resistance as a short-term reaction. The benefit of the integration of external evidence was questioned in the context of what healing means in primary care settings. GPs reflected on the opportunity to “heal” patients in a primary care setting in comparison to medical specialists’ settings not being enhanced by using the external evidence base of the digital intervention. Nevertheless, the interactions of the GPs show that they know about the severe effects of polypharmacy (“not only surgeons cut sharply,” GP_DD, p.23) and about the possibility that using the digital tool might enhance the quality of polypharmacy prescriptions. GPs discussed the implementation of the digital intervention in an orientation framework that refers to concepts of evidence-based medicine, adjusted to their professional experiences in primary care settings. Physicians share this common experience space, which is an indicator of a relevant dimension that is part of the primary care habitus.

The evolution of the focus group discussion shows that additional shared experience spaces exist and that various dimensions are layered in primary care habitus. To the extent that physicians belong to different common experience spaces (dimensions) and these reciprocally overlap, the habitus is multidimensional. Another important dimension that is represented in the narrations of physicians is the reasoning about ethical orientation regarding specific values like responsibility, avoiding patient harm, and codes of ethics for healing in primary care. Furthermore, a shared experience space was discovered regarding changing decision-making situations in the context of polypharmacy (subtopic 6).

Since GPs cope with polypharmacy in everyday practice, mostly concerning patients with chronic diseases, external evidence-based information is not perceived as very relevant for decision-making. Nevertheless, the integration of external evidence-based information into practice can become more relevant for GPs in cases where the condition of a patient with polypharmacy becomes acute and the patient requires urgent care as well as in ambiguous decision-making situations. In summary, the following three dimensions are included in the multidimensional habitus and reconstructed in the shared experience spaces of physicians: (1) relevance of pharmaceutical knowledge in shifting decision-making situations in polypharmacy; (2) medical code of ethics for clinical decision-making in the context of progressing digitalization; (3) concepts of evidence-based medicine based on professional experiences with polypharmacy in primary care settings.

### Results of documentary method analysis (primary care physicians’ habitus)

As we showed in the sequence analysis, three main dimensions of habitus were reconstructed (knowledge, ethics, professional experiences). We assume that the multidimensional habitus can lead to ambiguous behavioral outcomes regarding the acceptance of an intervention. The analyzed sequence contains descriptions and narrations leading to the conclusion that professional habitus may weaken or strengthen interventional effects. Because habitus is constituted during the professional socialization of physicians and is part of their professional identity, it is interpreted as a permanent characteristic of each physician that changes rather slowly—depending on physicians’ experiences during the implementation process. We found that physicians who discuss positive learning experiences and tend to base clinical decision-making on scientific evidence also describe themselves as motivated to use the intervention. In these examples, habitus functions as a facilitator of the implementation and can strengthen interventional effects. On the other hand, when the habitus favors resistance against integrating external evidence and an insistence on well-known practices, habitus functions as a barrier to implementation. In these cases, habitus weakens the motivation to adopt the intervention, and interventional effects on long-term outcomes are delayed.

### Results of the process-oriented model of implementation pathways

We aimed to synthesize results of content analysis and documentary method in the process-oriented model of implementation pathways and identify relevant and commonly shared topics among the two C-RCT groups related to

- Stages of behavior-related outcomes and
- The individual, social, and health care delivery level.

**Fig. 1:**
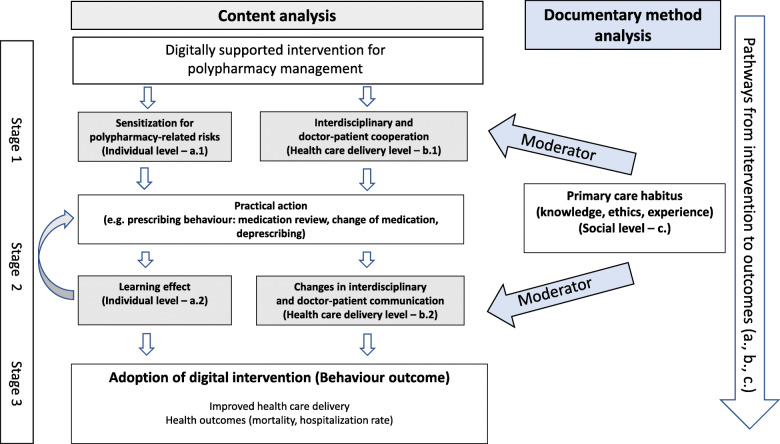
Process-oriented model of implementation pathways

Due to our process-related research focus, we allocated the results of content analysis to the different levels and stages in the implementation process. This approach gives us an overview of the subjectively experienced effects of the intervention as perceived by the physicians. In the context of content analytical result, the moderating influence of physicians’ habitus on adoption of the intervention is interpreted (Fig. 1). Scenarios of simple and complex pathways can be differentiated paradigmatically with increasing complexity (a–c):

- Simple pathway (positive behavioral outcomes): On the individual level, the digital intervention influences the participants’ cognitive experience of becoming sensitized to the risks associated with polypharmacy (stage 1), which leads to the practical action of changing their usual prescribing behavior. Adherence to the recommendations of the digital decision support system and the use of the pharmacological knowledge base results in a learning effect (stage 2). In a feedback loop, prescribing behavior changes sustainably, and the adoption of the intervention (stage 3)—as a regular tool in medical practice—is perceived as beneficial. The description of this pathway is informed by outcomes of content analysis on the individual level (a.1 and a.2).
- Complex pathways (unexpected behavioral outcomes): In the context of the digital transformation, physicians perceive a change in familiar forms of cooperation with pharmacists as experts in pharmacotherapy (health care delivery level). Physicians can digitally retrieve information and notes on pharmacotherapy. Additional information about other medical specialists involved in treatment is continuously available. As a result, overall transparency but also complexity in decision-making is increasing. Physicians seek orientation concerning mandatory ethical standards governing their professional responsibilities. The description of this pathway is informed by outcomes of content analysis on the health care delivery level (b.1 and b.2).
- Complex pathways (ambiguous behavioral outcomes): Primary care habitus functions as a moderator. It influences how the intervention affects short-, intermediate-, and long-term outcomes. Physicians question the benefits of using the pharmacological knowledge base of the intervention for clinical decision-making. Sensitization to polypharmacy-related risks through the use of the digital intervention is hindered, and learning effects are not experienced (individual level). The impact of risky prescribing behavior on patients’ well-being is trivialized by GPs, and information is not shared with patients (health-care delivery level). The usual prescribing practice is maintained (stage 2). The adoption of the intervention is delayed, depending on the level of the primary care habitus, until the benefits of its use are perceived (stage 3). The description of this pathway is informed by outcomes of content analysis and documentary method analysis (c.).

## Discussion

This study provides fresh insights into a specific aspect of the implementation process: the complexity of adoption behavior. Our findings show that in implementation science, the combination of qualitative methodological approaches like content analysis and documentary method analysis (habitus reconstruction), and visualization of implementation pathways contributes to the understanding of varying adoption behaviors.

Allocating content analytic results to different stages of behavior-related outcomes adds value to the identification of different pathways. In an empirical analysis, we were furthermore able to observe that physicians’ descriptions and narrations are related to experiences on the individual, health care-delivery, and social levels. Even though the habitus of physicians changes rather slowly and can weaken interventional effects promoting adoption, the analysis shows that physicians’ positive interaction experiences with the intervention might influence the change in habitus in the long term. The differentiation and visualization of topics in a model of implementation pathways help understand the complexity of adoption behavior. Furthermore, specific implications and policy strategies can be derived, depending on the addressed level.

In contrast to Straßner et al. [31], we found that on the individual level, physicians consider pharmacological information an essential dimension in the prescribing process in polypharmacy. They expect the digital availability of pharmacological knowledge to simplify decision-making. Also, physicians value the fact that recommendations made by the digital intervention can be used to enhance communication about medication changes with the patient. Referring to the digital expert system makes them feel more secure when explaining any initiated medication changes.

Our findings are consistent with previous research by Bauchner, who found that the complexity of clinical decision-making by physicians is embedded in a broad context of social norms [32]. As shown by Vogd, while evidence-based medicine aims to simplify the relationship between medical science and practice, it can instead burden it with more complexity [33]. Our study supports these findings since physicians mention their need for clear external evidence on which to base their decision-making but perceive the provided information as very complex for quick decision-making in practice. Physicians compared previous experiences with evidence-based guidelines with this digital intervention in a way that questioned the benefits of using it. Unexpected in this context was the physicians’ discussion of the orientation provided by ethical standards. They perceived the use of digital support to be associated with a change in professional and legal responsibilities.

Like Sinnige et al. [34], we found that physicians employ similar medication management strategies for polypharmacy, although there are variations in actual performance. Our findings also support the idea that physicians value decision support in polypharmacy, especially for geriatric and multimorbid patients. Unlike Sinnige et al., we did not find that physicians wanted the decision support option of meetings with pharmacists in which to discuss patients with complex problems. In our study, physicians perceived the digital intervention itself as a pharmacological expert system that processes patient data in a similar way as a pharmacist. It remains unclear if physicians experience the regular use of the digital intervention to replace the pharmacist, or whether an additional discussion is still needed.

Supporting the results of van de Velde et al. [8], our findings show that the adoption of the intervention critically depends on patient information being provided to physicians fast and automatically, rather than requiring a lengthy search. In Germany, a current (paper-based) medication plan has been mandatory for patients since 2016, and physicians expressed their expectation for this information to also be integrated automatically into the digital system.

Our findings have important implications for upscaling the intervention: Physicians perceive their behaviour to become more transparent through the digitalization of the prescribing process. Therefore, implemented evidence-based tools must be as transparent as possible concerning their database and underlying calculations. Increased transparency is a goal to encourage users to routinely use the intervention and accept the related workload during the implementation process.

Clinical decision-making processes in polypharmacy are influenced by the intervention on several levels that influence each other. It has been shown that there is an individual need for support in the field of polypharmacy, but the adoption of the intervention is strongly influenced by the social environment of the doctors. The professional role of GPs, which is reflected in the habitus of general practitioners and their socialization, is evidence of how strongly the social environment influences the doctors’ practical actions in the prescription process and thus the adoption of the intervention. The intervention also influences and changes social relationships in the clinical decision-making process (doctor-patient, interprofessional cooperation), with doctors reporting reassurance in the prescription process while using the intervention, even though the changes in inter-professional cooperation caused by the intervention and their influence on the quality of prescriptions merit further study.

Physicians in the intervention group may change their prescribing behavior and prioritization of important aspects of clinical decision-making when prescribing polypharmacy - in terms of examining the needs of the individual patient, scientific evidence, and medical experience. Positive interaction experiences with the intervention are associated with physicians’ perceptions of an increased risk-awareness and behavioral intention to base clinical decision-making on polypharmacy prescribing on scientific evidence. In comparison, we have found that clinical decision-making on polypharmacy without digital support is associated with great uncertainty. Physicians have developed habitual strategies to compensate for these uncertainties in practice but have expressed a need for new practical approaches to the management of polypharmacy.

### Strengths and limitations

The findings of this study reflect the opinions of 27 primary care physicians and provide an in-depth understanding of the GPs’ expectations and interactions with the digital intervention. A purposive sampling strategy was planned to be conducted, but we were unable to choose participants exclusively by theoretical characteristics. A pragmatic decision was made to apply a convenient sampling strategy with a purposive aim to collect data from GPs of both C-RCT groups. Data analysis used qualitative data collected in the first year of implementation, so only physicians who were enrolled during that period had the opportunity to participate in our study. It can be assumed that early adopters, who participated in this initial phase of the project, are more engaged and motivated to adopt the intervention. Despite their overall interest in the project, this sample still expressed relatively stable concerns about the adoption of the system. The implication is that although over time, higher numbers of physicians are going to use the intervention, this development will not reliably directly result in routine uptake of the digital intervention in practice.

## Conclusion

German physicians experience positive effects and increasing polypharmacy-related risk awareness while applying the digital intervention. They expect the digital expert system to provide reassurance during prescribing processes and benefit their communication with patients concerning medication management. However, they have not yet routinely adopted the intervention.

Physicians are relatively open to change processes in polypharmacy management. In the short term, the intervention sensitizes physicians to polypharmacy-related risks. The intervention also affects interdisciplinary and doctor-patient communication. Therefore, adoption of the digital intervention, behavior changes, and transformation of physicians’ habitus are anticipated in the intermediate and long term. To ensure uptake, it is necessary to address the above-mentioned implications, such as by promoting (1) facilitated positive learning experiences, (2) simplified evidence-based information, and (3) a clarified professional code of ethics and responsibilities. Variations in actual performance and use of the digital intervention are moderated by the physicians’ multidimensional habitus (knowledge, ethics, experience). The analysis of the moderating influence of physicians’ habitus adds evidence in explaining variations in the effectiveness of digital interventions on health-related outcomes.

## Supplementary information

**Additional file 1.** COREQ (COnsolidated criteria for REporting Qualitative research) Checklist.

**Additional file 2.** The TIDieR (Template for Intervention Description and Replication) Checklist*.

**Additional file 3.** Stages of behavior-related outcomes of the digital intervention.

## Data Availability

No datasets are available from this study due to participant consent restricting data use to the research team.

## Abbreviations

TiDIER: Template for Intervention Description and Replication
COREQ: Consolidated criteria for reporting qualitative research
