# Supplementary material for: From sensitization to adoption? A qualitative study of the implementation of a digitally supported intervention for clinical decision making in polypharmacy
Source: Implement Sci. 2020 Sep 21;15:82. doi: 10.1186/s13012-020-01043-6 (PMC7507604; doi:10.1186/s13012-020-01043-6)
Supplement: Supplementary file 2 — Additional file 2. The TIDieR (Template for Intervention Description and Replication) Checklist*. [file 13012_2020_1043_MOESM2_ESM.pdf]

TIDieR

Template for Intervention Description and Replication

The TIDieR (Template for Intervention Description and Replication) Checklist*:

Information to include when describing an intervention and the location of the information

Item number	Item	Where located ** Primary paper (page or appendix number)	Other † (details)
	**BRIEF NAME**		
**1.**	Provide the name or a phrase that describes the intervention.	Application for a digitally supported Medication Management Support System (AdAM)	
	**WHY**		
**2.**	Describe any rationale, theory, or goal of the elements essential to the intervention.	By providing drug-therapy-relevant patient information to the general practitioners in the intervention group via a digitally supported application, the quality and safety of prescribing for patients with polypharmacy should be improved (e.g. decrease of potentially inappropriate medication, adverse drug events).	
	**WHAT**	The digitalized decision support system can be used by general practitioners in the intervention group via personal access.	https://clinicaltrials.gov/ct2/show/NCT03430336

		GPs in the wait-list control group provide usual care. The decision support system provides drug-therapy relevant information of patients with polypharmacy. Other features are: modification of medication, assessment of medication appropriateness; medication plan; guidance in medication process)	
**3.**	Materials: Describe any physical or informational materials used in the intervention, including those provided to participants or used in intervention delivery or in training of intervention providers. Provide information on where the materials can be accessed (e.g. online appendix, URL).	General practitioners can use the provided drug-therapy relevant information to modify medication and generate an up-to-date medication plan and hand it out to their patients.	https://www.kvwl.de/arzt/ivf/innov_fonds/adam_infoblatt.pdf<br><br>https://www.kvwl.de/arzt/ivf/innov_fonds/adam_kurzinfo_versorgungsvertrag.pdf
**4.**	Procedures: Describe each of the procedures, activities, and/or processes used in the intervention, including any enabling or support activities.	After physicians register for participation in the study, the Association of Statutory Health Insurance Physicians (KVWL) provides telephone support and training to use the digital application for general practitioners and their medical assistants in the intervention group. Training includes an	

		one hour frontal teaching for the use of the digital application with additional information material. Via an online platform, training and information material can also be used by the GPs of the intervention group. Telephone support and training is provided on a voluntary basis.	
	**WHO PROVIDED**		
**5.**	For each category of intervention provider (e.g. psychologist, nursing assistant), describe their expertise, background and any specific training given.	The Health Insurance Company (BARMER) and the Association of Statutory Health Insurance Physicians (KVWL) are responsible for the delivery of the intervention to general practitioners and patients. Pharmacists or pharmaceutical technical assistants employed by KVWL provide training and support. Telephone support is provided by trained employees of KVWL via a telephone hotline.	
	**HOW**		
**6.**	Describe the modes of delivery (e.g. face-to-face or by some other mechanism, such as internet or telephone) of the intervention and whether it was provided individually or in a group.	The digital application is delivered via internet and personalized access	

		on the computer of general practitioners in the intervention group. Training and support are provided face-to-face (individually and in groups) or in general practices and via telephone.	
	**WHERE**		
**7.**	Describe the type(s) of location(s) where the intervention occurred, including any necessary infrastructure or relevant features.	The intervention is delivered to the registered general practices of the intervention group. Information is retrieved user-initiated.	Internet access
	**WHEN and HOW MUCH**		
**8.**	Describe the number of times the intervention was delivered and over what period of time including the number of sessions, their schedule, and their duration, intensity or dose.	Assessment of medication appropriateness should be conducted at least one time per year for each included patient. Depending on physicians' demand, the assessment can also be carried out more often.	01.01.2018 – 31.12.2020
	**TAILORING**		
**9.**	If the intervention was planned to be personalised, titrated or adapted, then describe what, why, when, and how.	N/A	______________
	**MODIFICATIONS**		
**10.‡**	If the intervention was modified during the course of the study, describe the changes (what, why, when, and how).	N/A	______________
	**HOW WELL**		

**11.**	Planned: If intervention adherence or fidelity was assessed, describe how and by whom, and if any strategies were used to maintain or improve fidelity, describe them.	To measure physicians' adherence, medication appropriateness is monitored throughout the study.	Evaluators from the participating universities in the project AdAM (see https://clinicaltrials.gov/ct2/show/NCT03430336
**12.‡**	Actual: If intervention adherence or fidelity was assessed, describe the extent to which the intervention was delivered as planned.	Not yet applicable	

** **Authors** - use N/A if an item is not applicable for the intervention being described. **Reviewers** – use '?' if information about the element is not reported/not sufficiently reported.

† If the information is not provided in the primary paper, give details of where this information is available. This may include locations such as a published protocol or other published papers (provide citation details) or a website (provide the URL).

‡ If completing the TIDieR checklist for a protocol, these items are not relevant to the protocol and cannot be described until the study is complete.

* We strongly recommend using this checklist in conjunction with the TIDieR guide (see *BMJ* 2014;348:g1687) which contains an explanation and elaboration for each item.

* The focus of TIDieR is on reporting details of the intervention elements (and where relevant, comparison elements) of a study. Other elements and methodological features of studies are covered by other reporting statements and checklists and have not been duplicated as part of the TIDieR checklist. When a **randomised trial** is being reported, the TIDieR checklist should be used in conjunction with the CONSORT statement (see www.consort-statement.org) as an extension of **Item 5 of the CONSORT 2010 Statement.** When a **clinical trial protocol** is being reported, the TIDieR checklist should be used in conjunction with the SPIRIT statement as an extension of **Item 11 of the SPIRIT 2013 Statement** (see www.spirit-statement.org). For alternate study designs, TIDieR can be used in conjunction with the appropriate checklist for that study design (see www.equator-network.org).
