# Supplementary material for: From sensitization to adoption? A qualitative study of the implementation of a digitally supported intervention for clinical decision making in polypharmacy
Source: Implement Sci. 2020 Sep 21;15:82. doi: 10.1186/s13012-020-01043-6 (PMC7507604; doi:10.1186/s13012-020-01043-6)
Supplement: Supplementary file 3 — Additional file 3. Stages of behavior-related outcomes of the digital intervention. [file 13012_2020_1043_MOESM3_ESM.pdf]

Additional File 3 Stages of behavior-related outcomes of the digital intervention

Stage 1	Interviews (intervention group)	Focus groups (intervention group)	Focus groups (wait list control group)
Sensitization for risks related to polypharmacy (individual level)	“Yes, the necessity of the interaction check in polypharmacy, especially for our geriatric patients. That is, just when psychiatric medications are added, which have unknown indications, that one ultimately no longer has in mind what interacts with each other in what way. And that simply gives one the security then to carry out this analysis. The thing is, we have more and more chronically ill, old patients with a lot of drugs, that this also ... and that is increasing. And that is why I believe it is increasingly necessary to have this feeling of security that everything is going well concerning polypharmacy.” [GP1, p.3]<br><br>“It suddenly comes to unclear laboratory values which you cannot explain, and then it is, of course, interesting to know, are there possibly other drugs. Or are there other doctors involved that you don't know about.” [GP 8, p.5]	“As I said, you get a little more sensitive about the interactions, especially when it comes to specialist medication that you often don't have on your radar. Well, I always try to include them in the medication plan and then write behind it, neurologist, gynecologist, or something else, but we don't get any reports from the gynecologists, yes. If the patients don't tell us that they are getting the medication, then we don't know that either. Also, with the neurological patients, with some of them, I was surprised by what they take on the side.” [FG4, GP_CC, p.14]<br><br>“And the advantage of this system, or this program in general, is that I have reviewed the patients who are now enrolled […] that I looked again on the medication plan and see, does he still take everything that I have there now, or does he already take more.” [FG4, GP_AA, p.10]	“Well, I'd like an overview of the actual medications taken by each patient. From other colleagues, or even what he might get in the pharmacy. Then I would like professional assistance with the assessment of interactions, side effects, contraindications, and which of the drugs are suitable for old people at all, and which tend not to be.” [FG1, GP_CC, p.3]<br><br>“I think it's good that polypharmacy is coming into focus. That doctors are sensitized to it, or that everyone, everyone is sensitized to it, and patients are also sensitized to it, and it is still a bit difficult to really get down from ten to five [drugs], I don't always see myself in a position to do that, but I think it is important to be more involved than in the past ten years. And the goal is really, yes, maybe less is more.” [FG2, GP_DD. p.24]
Interdisciplinary and doctor-patient cooperation (health care delivery)	“So now you get, practically all the time, calls from pharmacists who think something is not working with one or the other, but they don't see the clinical presentation. Now, if you have a Parkinson's patient and want to calm him down somehow because he's nervous all night, then maybe that reduces the effect of his Parkinson's medication, but then from the pharmaceutical perspective alone, that's not seen. The medical assistant sits there, a red light goes on, and they tell the patient, watch out, this reduces the effect of the Parkinson medication; then a relative comes by and says, you wrote down something that possibly reduces the effect of the Parkinson medication, we cannot take that. What impression does that make? If then ... that's what it is, if there are a lot of people interfering, that's bullshit. Somebody has to say how it works and then it's okay.” [GP6, p.8]	“I didn't have that experience, of course, but... well, that's new to me. I know it otherwise, as I said, also from the pharmacists, because I constantly or conveniently get information from them, like there is an incompatibility with azithromycin or something else. But where we have a comprehensive medication list from all kinds of doctors who have treated the patient, that has not yet existed.” [FG3, GP_BB, p.8]<br><br>„Where is the sense and purpose [using the digital tool]? What is the whole thing supposed to do ... what is important information? Where do I perhaps not need to look like that? Do I only perceive it, the specialist medication or should I integrate it into the system? [FG3, GP_AA, p.13]	“I think if you participate in such projects, you also have the chance to work better with patients, nursing services, with colleagues or sometimes with hospitals. So that you call back and say, is that really the case? Can't you change one or the other or don't always add the next one? Another specialist and another specialist, the urologist and the cardiologist and the hospital, then again and then the nursing service with a proposal. Well, I think it has an important control function. So, not in a negative sense, but in a very positive sense.” [FG1, GP_EE, p.4]

	"So, they [patients] feel safer and also, I think, more confident about why they take something. Because you can explain what the tablets are really good for." [GP7, p.4]		
Stage 2	Interviews (intervention group)	Focus groups (intervention group)	Focus groups (wait-list control group)
Learning effect (individual level)	"I like to use it [digital tool] and see also a lot of sense in it, because I also learn again, refresh again, knowledge that is perhaps still present somewhere in the back of my mind, but to update this again, but I find this information very good. When it comes to dosage, for example in the case of kidney failure, oh yes, aha, right, of course ... you could think about it or just recommend something to implement, so I think that's very good. It makes my work as a doctor much easier when prescribing, so I think that makes a lot of sense." [GP1, p.6]<br><br>"It's okay to pay more attention, but I... on the basis of this, yes, the recommendation I can ... I will still not change the therapy because this is also from the cardiologist and this is the treatment for heart failure, yes, even if the side effects or the interactions are known." [GP2, p.3]	"It's always one of those little training sessions you do. Other patients also benefit from it because I suddenly see that, oh, these medications don't go together so well after all." [FG4, GP_AA, p.33]<br><br>"I now find myself with my patients, well, coming to their routine visits, simply perceiving these risks more intensely and then changing it, yes, with the other patients as well, if I consider it initiated. And I found that, for example, quite good." [FG3, GP_DD, p.9]	"And at the moment, when a project like AdAM is running, we can, of course, say, okay, we've had it reviewed externally again. Someone looks at it again, and of course, we are a bit more on the safe side from a legal point of view. Of course, our responsibility is still to give or not to give medication at all, but we can at least say what the medication is like, what the consequences are and that interactions have been checked externally." [FG2, GP_DD, p.6]
Changes in interdisciplinary and doctor-patient communication (health care delivery)	"You could see right away [using the digital tool], okay, he got two different ones within one quarter, that was a bit strange. Then I talked to a neurologist on the phone, where several drugs were administered that change the QT time. For things like that it was good. Nothing happened, but, well, something could have happened." [GP5, p.8]<br><br>"It's good, especially for the patients, they all saw great sense in it and found it good. So, I did that mostly in the presence of the patients, so they immediately saw what kind of information there was about interactions." [GP1, p.2]	"I have patients where the medication just did not really fit and where I can exchange views with the specialists, who are also named [in the digital tool], where patients are being treated. Well, I think that's quite good. [FG4, GP_CC, p.5]<br><br>"Such prescription chains are created, and I believe that these chains cannot be broken by specialists because they think too narrowly. And we as general practitioners, we have to try to break them up again with such instruments [digital tool]." [FG3, GP_AA, p.10]	"Yes, and of course, that is also where you would get a bit of support with software that recognizes things in a structured way, as long as you have to rely on your knowledge, on what you try to achieve through extensive further training and the like. The pharmacist is, of course, a very important interface, especially since pharmacists already have better software products at their disposal than we have in our practices, in terms of interactions, for example." [FG2, GP_EE, p.5]

[GP= General practitioner, FG= Focus Group, AA-EE = synonyms for GPs in FGs]
